# Supplementary material for: MEIS1 in Hematopoiesis and Cancer. How MEIS1-PBX Interaction Can Be Used in Therapy
Source: J Dev Biol. 2021 Oct 13;9(4):44. doi: 10.3390/jdb9040044 (PMC8544432; doi:10.3390/jdb9040044)
Supplement: Supplementary file 1 [file jdb-09-00044-s001.zip › jdb-1394886-supplementary.pdf]

SUPPLEMENTARY MATERIAL

Material and Methods

Cloning and Mutagenesis

For cloning MEIS1-GFP into the retroviral vector pBABE, we used a human MEIS1 template. Our cloning strategy consisted of replacing PREP1-GFP previously produced [1] with MEIS1-GFP in the pBABE vector, but as no suitable restriction sites were available for straight replacement of PREP1 with MEIS1 before the GFP insertion, we had to proceed stepwise, by using the overlap PCR method [2]. Using as cloning restriction sites SnaBI and SalI, we designed four primers (see **Supplementary Table S1**): the SnaBI and the SalI restriction sites were placed respectively in the MEIS1 forward (MEIS1 SnF) and in the GFP reverse primers (GFP SAR RV).

Supplementary Table S1: primers used for cloning MEIS1-GFP wild-type into pBABE vector

Primer Name	Sequence
MEIS1 SnF FW	CGTACGTAGCCACCATGGCGCAAA
GFP SaR RV	GCGTCGACTTACTTGTACAGCTCGTCCA
MEIS GFP FW	ACTACATGATGGTACCGCGGGCC
MEIS GFP RV	GGCCCGCGGTACCATCATGTAGT

Then, to fuse MEIS1 with GFP, we used two internal overlapping primers, both containing the C-terminal end of MEIS1 and an N-terminal portion of the GFP sequences. MEIS1 L83/L86/L87/I90 and L149/I152/L156/L159 genes were synthetized by GenScript and replaced the wild-type MEIS1 through SnaBI/SalI. DNA sequencing confirmed the presence of the correct MEIS1-GFP insertions and position into the pBABE vector.

Cell Culture and Retroviral Infection

Immortalized mouse embryonic fibroblasts (MEFS) were cultured in DMEM (Lonza)+ 10% FBS + 2 mM L-glutamine. For the retroviral transduction, Phoenix-Ampho cells were transiently transfected with the retroviral vectors pBABE-puro using standard calcium-phosphate procedure. Cell supernatants containing retroviruses were filtered using a 0.45 µm filter and used to perform two cycles of infection. Two days after infection, cells were put in selection by supplementing the culture medium with puromycin (1,4 µg/mL). To check for the expression of the MEIS1-GFP-constructs, 5 days post-infection MEFs cell lines were assayed by western blot analysis by loading 30 µg of total cellular lysate using anti-GFP antibody (Abcam #Ab290) and by flow-cytometry analysis (**Supplementary Figure S1**).

For flow cytometry a FACSCalibur (Becton Dickinson) instrument was used, and the data were analyzed with Kaluza Analysis Software (Backman Coulter).

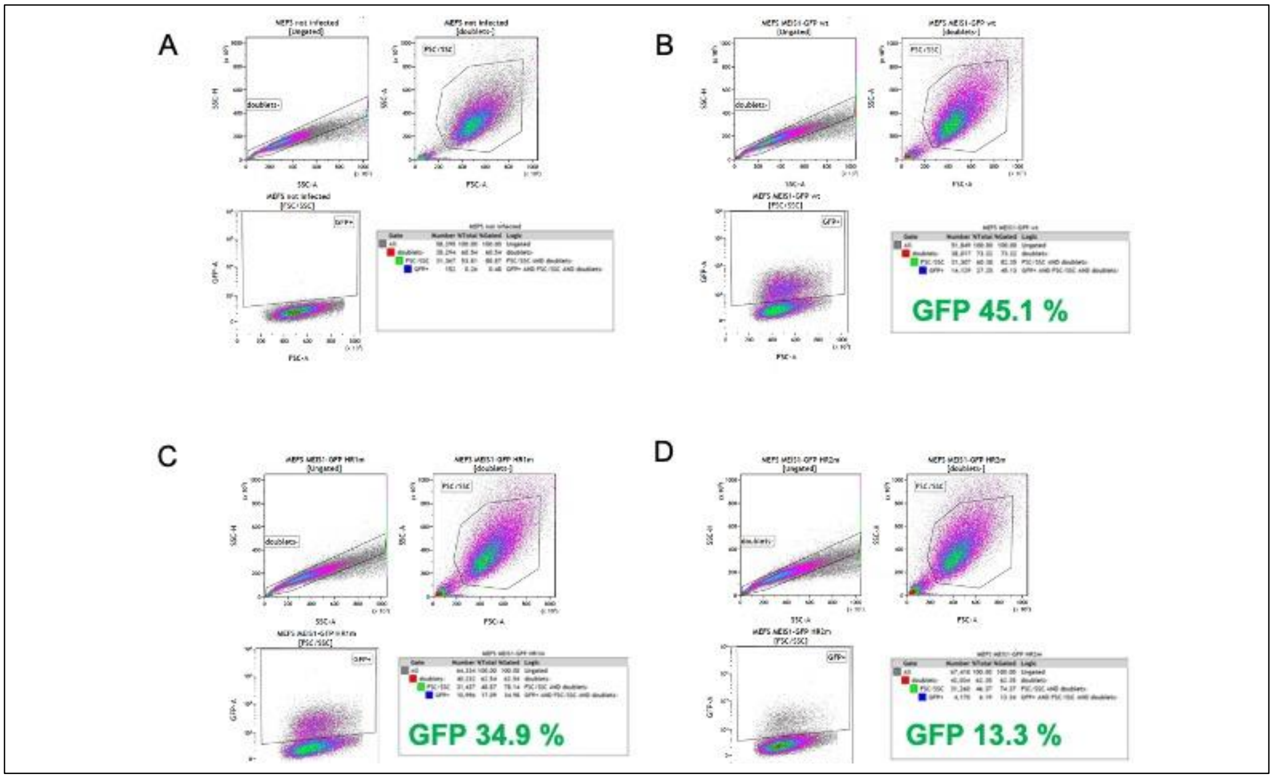

Supplementary Figure S1: Flow-cytometry analysis of MEFs expressing wild-type and mutant MESI1-GFP fusion proteins used for the pull-down and for the immunofluorescence experiments. In Panel A is shown the control of not infected MEFs, with representative gating strategy: single cells were chosen for analysis after doublet discrimination, and GFP-positive cells were detected. MEFs retrovirally expressing wild-type MEIS1-GFP are shown in Panel B; MEIS1-GFP HR1m (L83/L86/L87/I90) in Panel C; and MEIS1-GFP HR2m (L149/I152/L156/L159) in Panel D. In the figure is also indicated the percentage of cells expressing MEIS1-GFP wild-type and mutants.

Confocal Microscopy Acquisition and Images Analysis

Confocal microscopy acquisition was performed on a Leica TCS SP8 laser-scanning confocal microscope. For image acquisition, an HC PL APO CS2 63X/1.40 oil immersion objective was used. The microscope was controlled by software Leica Application Suite X. Images were acquired with format 1024x1024 pixels and 2x optical zoom. The plugin adapted for Fiji [3] recognizes the nuclei on the DAPI channel using Otsu’s thresholding algorithm [4], upon noise removal [5] and application of a gaussian filter. Then after background removal, the mean intensity of the GFP signal is extracted. For each cell, the cytoplasmic mean intensity is obtained from a drawing by hand region, based on the profile defined by the Phalloidin-TRITC staining (Sigma-Aldrich, # P1951). Finally, the plugin reports the ratio between the nuclear and the cytoplasmic signal for each cell.

Pull-Down of PBX1 in MEFs Cells

For the pull-down assay, MEFs overexpressing wild-type MEIS1-GFP, or the mutants were grown in a 10 cm plate to 70% confluence, then lysated in 1 ml of TEB_{150} extraction buffer (50 mM HEPES pH 7.3, 150 mM NaCl, 2 mM MgCl2, 5 mM EGTA, 0.5% Triton X-100, 10% Glycerol, protease inhibitors), and quantified using the BCA kit (Thermo Scientific). 1 mg of lysate for each condition was then incubated with 35 µl of GFP-trap slurry (Chromotech GFP-Trap_M), previously equilibrated with TEB_{150} buffer, for 1 h at 25 C

under rotation. After incubation, beads were washed three times with TEB_{150} buffer and then boiled with sample buffer, before loading into 8% SDS PAGE. The membrane was transferred using the semi-dry Invitrogen system and then blocked for 1 h in TBS-T (TBS-T: 500 mM NaCl, 20 mM Tris–Cl, pH 7.5, 0.05 % (w/v) Tween 20) with 5% milk. The membrane was incubated under gentle agitation with primary antibody overnight at 4 C (see **Supplementary Table S2** for details). Secondary goat anti-mouse HRP Ab (Bio-Rad) (1:10000 dilution) or goat anti-rabbit HRP Ab (Bio-Rad) (1:10000 dilution) were added in blocking buffer and incubated for 1 h with gentle agitation at room temperature. Between incubation with the different primary antibodies, the membrane was stripped with Stripping buffer (Restore Western blot, Thermo scientific). Image acquisition of the membranes was done with ChemiDoc imager (Bio-Rad), and quantification of the results was performed using the ImageLab software version 6.1 (Bio-Rad).

Supplementary Table S2: antibodies used for the detection of the pull-down assay

Antibody	Dilution in 5% Milk	Brand and Catalog Number
Rabbit polyclonal Anti-PBX1	1:2000	Cell Signaling #4342
Rabbit polyclonal Anti-GFP	1:2000	Abcam #290
Mouse monoclonal Anti-vinculin	1:5000	Sigma #V-9131

References

1. Bruckmann, C.; Tamburri, S.; De Lorenzi, V.; Doti, N.; Monti, A.; Mathiasen, L.; Cattaneo, A.; Ruvo, M.; Bachi, A.; Blasi, F. Mapping the native interaction surfaces of PREP1 with PBX1 by cross-linking mass-spectrometry and mutagenesis. *Sci. Rep.* **2020**, *10*, 16809, doi:10.1038/s41598-020-74032-w.
2. Bryksin, A.V.; Matsumura, I. Overlap extension PCR cloning: a simple and reliable way to create recombinant plasmids. *Bio.Techniques.* **2010**, *48*, 463-465, doi:10.2144/000113418.
3. Schindelin, J.; Arganda-Carreras, I.; Frise, E.; Kaynig, V.; Longair, M.; Pietzsch, T.; Preibisch, S.; Rueden, C.; Saalfeld, S.; Schmid, B.; et al. Fiji: an open-source platform for biological-image analysis. *Nat. methods* **2012**, *9*, 676-682, doi:10.1038/nmeth.2019.
4. ImageJ Auto Treshold Otsu. Available online: https://imagej.net/plugins/auto-threshold#Otsu (accessed on 15 June 2021)
5. ImageJ Rolling Ball Background Subtraction. Available online: https://imagej.net/plugins/rolling-ball-background-subtraction (accessed on 15 June 2021)
